# Development of Novel Lightweight Al-Rich Quinary Medium-Entropy Alloys with High Strength and Ductility

**DOI:** 10.3390/ma14154223

**Published:** 2021-07-28

**Authors:** Po-Sung Chen, Yu-Chin Liao, Yen-Ting Lin, Pei-Hua Tsai, Jason S. C. Jang, Ker-Chang Hsieh, Chih-Yen Chen, Jacob C. Huang, Hsin-Jay Wu, I-Yu Tsao

**Affiliations:** 1 Institute of Material Science and Engineering, National Central University, Taoyuan 320, Taiwan; thepacific999@gmail.com (P.-S.C.); peggyphtsai@gmail.com (P.-H.T.); evauseonly@gmail.com (I.-Y.T.); 2 Department of Mechanical Engineering, National Central University, Taoyuan 320, Taiwan; llllurker@gmail.com (Y.-C.L.); abcdan1991@gmail.com (Y.-T.L.); 3 Department of Materials and Optoelectronic Science, National Sun Yat-sen University, Kaohsiung 804, Taiwan; khsieh@mail.nsysu.edu.tw (K.-C.H.); cychen@mail.nsysu.edu.tw (C.-Y.C.); chihuang@cityu.edu.hk (J.C.H.); 4 Department of Materials Science & Engineering, Hong Kong Institute for Advanced Study, City University of Hong Kong, Kowloon 999077, Hong Kong; 5 Department of Materials Science and Engineering, National Yang Ming Chiao Tung University, Hsinchu 300, Taiwan; ssky0211@nctu.edu.tw

**Keywords:** medium-entropy alloy, lightweight, nonequiatomic, dual phase, heat treatment

## Abstract

Most high-entropy alloys and medium-entropy alloys (MEAs) possess outstanding mechanical properties. In this study, a series of lightweight nonequiatomic Al_50_–Ti–Cr–Mn–V MEAs with a dual phase were produced through arc melting and drop casting. These cast alloys were composed of body-centered cubic and face-centered cubic phases. The density of all investigated MEAs was less than 5 g/cm^3^ in order to meet energy and transportation industry requirements. The effect of each element on the microstructure evolution and mechanical properties of these MEAs was investigated. All the MEAs demonstrated outstanding compressive strength, with no fractures observed after a compressive strain of 20%. Following the fine-tuning of the alloy composition, the Al_50_Ti_20_Cr_10_Mn_15_V_5_ MEA exhibited the most compressive strength (~1800 MPa) and ductility (~34%). A significant improvement in the mechanical compressive properties was achieved (strength of ~2000 MPa, strain of ~40%) after annealing (at 1000 °C for 0.5 h) and oil-quenching. With its extremely high specific compressive strength (452 MPa·g/cm^3^) and ductility, the lightweight Al_50_Ti_20_Cr_10_Mn_15_V_5_ MEA demonstrates good potential for energy or transportation applications in the future.

## 1. Introduction

Since the Iron Age, metallic materials have played a major role in the development of human civilization [1]. In 1996, high-entropy alloys (HEAs) were proposed [2], characterized by the multiple main elements in their design. HEAs are composed of multiprinciple elements which possess unique properties such as high entropy, lattice distortion, sluggish diffusion, and cocktail effects [3,4]. Due to these characteristics, HEAs have better mechanical properties than traditional alloys. However, despite the advantageous properties of HEAs, their density typically exceeds 10 g/cm^3^ [5,6], which severely limits their application in the transportation and energy industries.

In recent years, a nonequiatiomic alloy design has been proposed [7] that not only retains the characteristics of the existing HEAs but also increases the flexibility of the HEA design [8]. In this regard, design is no longer limited to high entropy but has developed toward medium entropy. With one main element accounting for approximately 50–70% of an alloy and the addition of other elements, the resultant structure can maintain a solid solution structure [9,10]. These developments have further expanded the range of alloy designs [11,12].

Some lightweight equiatiomic high-entropy alloys (LWHEAs) composed of light metals such as Al and Ti have been studied [13]. However, the intermetallic compounds composed of Al and Ti always adversely affect the mechanical properties of the LWHEAs [14,15,16]. Therefore, a simple-phase microstructure is essential to obtain the desired mechanical properties required for promising future applications.

In this study, nonequiatomic lightweight Al_50_–Ti–Cr–Mn–V MEAs were designed to achieve low density with well-balanced strength–ductility synergy. A high content of Al was selected to form lightweight MEAs (density defined as <5 g/cm^3^), and the influence of the contents of Ti, Cr, Mn, and V on the MEAs microstructure and mechanical properties was evaluated. Subsequently, the characteristics of the elements obtained through the experiments were further adjusted to an optimal ratio of Ti, Cr, Mn, and V in the MEAs to achieve advantageous mechanical properties.

## 2. Materials and Methods

### 2.1. Materials

Al (purity 99.99%), Ti (purity 99.99%), Cr (purity 99.99%), Mn (purity 99.9%), and V (purity 99.9%) were the raw materials used to form the lightweight nonequiatiomic Al_50_–Ti–Cr–Mn–V MEAs. The ingots were prepared through arc melting under an Argon gas atmosphere. All ingots were remelted four times to ensure homogeneity. The samples were fabricated through drop casting with dimensions of 35 mm × 30 mm × 5 mm.

### 2.2. Microstructure Characterization

The density of the MEAs was measured using Archimedes’ principle. X-ray diffraction (XRD; D2 Bruker, Billerica, MA, USA) was used to identify the crystal structure of the MEAs with Cu K_α_ radiation. Scanning electron microscopy (SEM; F50 Inspect, FEI, Hillsboro, OR, USA) with energy dispersive spectroscopy was employed to characterize the microstructure of the MEAs.

### 2.3. Mechanical Testing

A Vickers hardness testing machine (HV-100 Mitutoyo, Kawasaki, Japan) was used to measure the hardness of the MEA samples with a loading of 5 kg for 10 s and five readings were measured at random areas in the specimen. A universal testing machine (HT9102 Hung Ta, Taichung, Taiwan) was used to conduct compression testing under quasistatic loading with a strain rate 1 × 10^−4^/s at room temperature. The dimension of the compression testing samples was fabricated as a cylinders of Ø 3 mm × 6 mm and three readings were measured.

## 3. Results and Discussion

### 3.1. Density of the Al_50_–Ti–Cr–Mn–V MEAs

**Figure 1:**
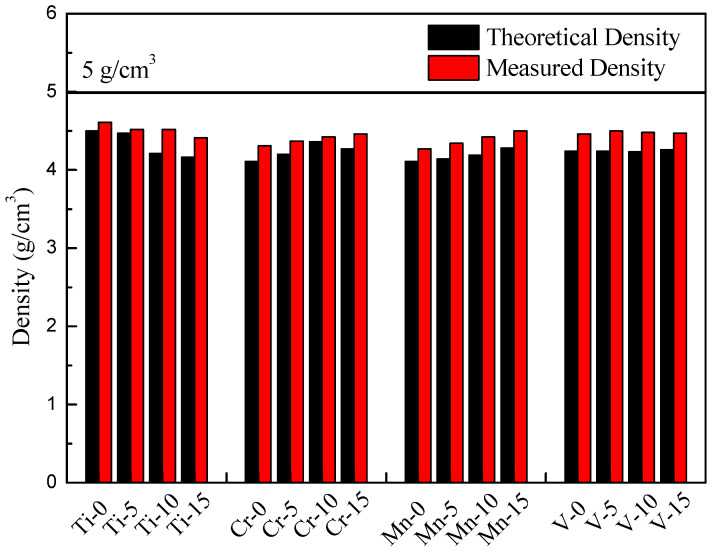
Density of the Al_50_–Ti–Cr–Mn–V alloys.

By maintaining the atomic ratio of Al at 50%, Al_50_–Ti–Cr–Mn–V MEAs can achieve low density (<5 g/cm^3^), with a density distribution from 4 to 5 g/cm^3^. By using Archimedes’ principle, all measured results were close to the theoretical results by mixing rules; the theoretical and measured densities are presented in Figure 1.

### 3.2. Optimization of the Mechanical Properties of the Al_50_–Ti–Cr–Mn–V MEAs

**Figure 2:**
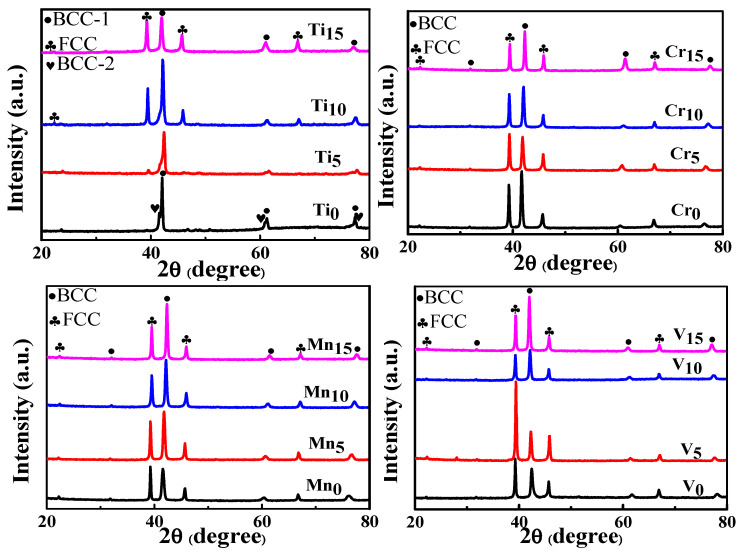
XRD patterns of the Al_50_–Ti–Cr–Mn–V alloys.

**Figure 3:**
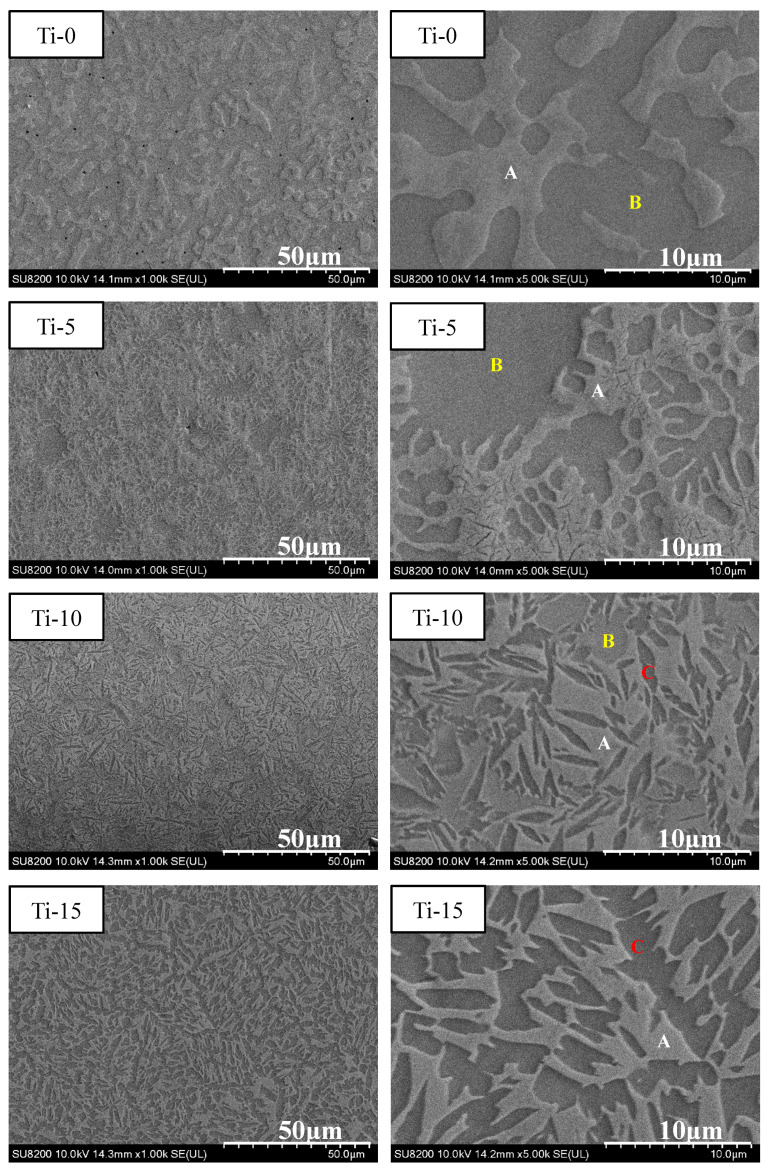
SEM images of the Al_50_(CrMnV)_50-x_Ti_x_ (x = 0, 5, 10, 15) alloys (A: BCC-1; B: BCC-2; C: FCC).

**Figure 4:**
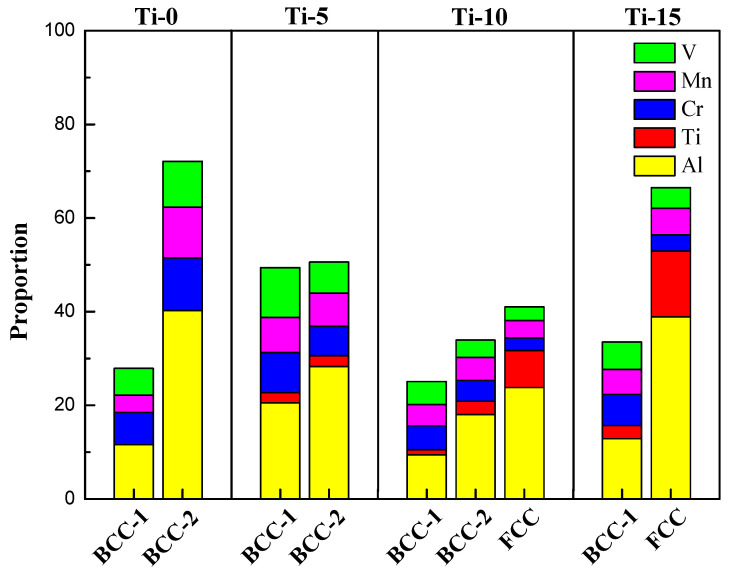
The proportion of Al, Ti, Cr, Mn and V in the dual-phase of Al_50_–Ti–Cr–Mn–V alloys.

**Figure 5:**
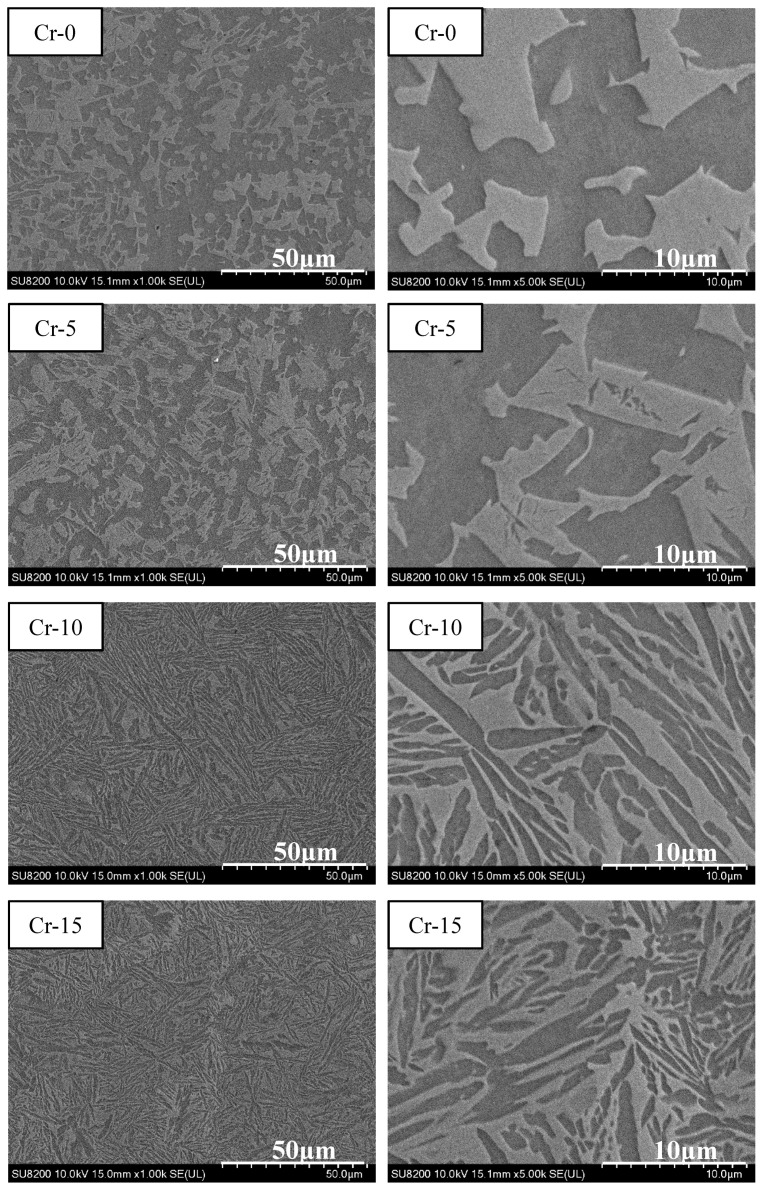
SEM images of the Al_50_(TiMnV)_50-x_Cr_x_ (x = 0, 5, 10, 15) alloys.

**Figure 6:**
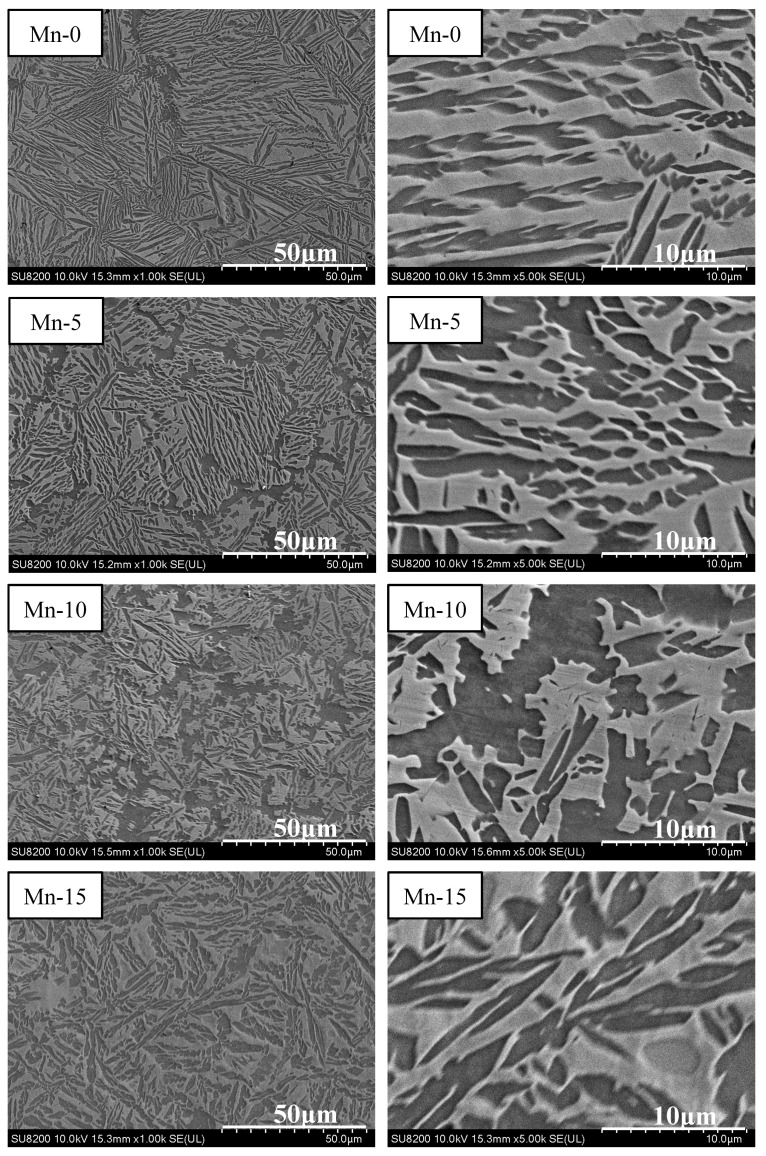
SEM images of the Al_50_(TiCrV)_50-x_Mn_x_ (x = 0, 5, 10, 15) alloys.

**Figure 7:**
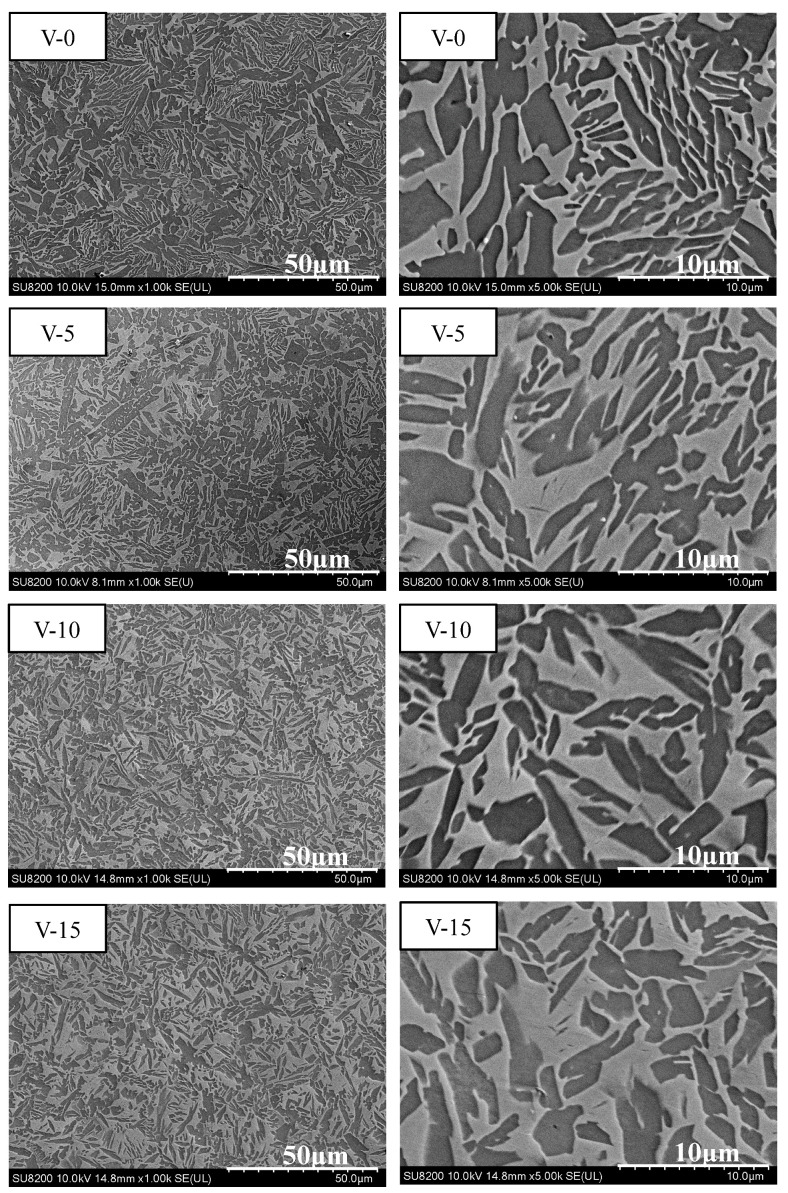
SEM images of the Al_50_(TiCrMn)_50-x_V_x_ (x = 0, 5, 10, 15) alloys.

Based on the mechanical compression results and phase morphology analysis from the XRD patterns and SEM images, the effects of adding the elements Ti, Cr, Mn, and V to the Al_50_(CrMnV)_50-x_Ti_x_, Al_50_(TiMnV)_50-x_Cr_x_, Al_50_(TiCrV)_50-x_Mn_x_, and Al_50_(TiCrMn)_50-x_V_x_ (x = 0, 5, 10, 15) quinary MEAs were investigated. An increase in the Ti ratio induced phase transformation from the body-centered cubic (BCC)-1 and BCC-2 phase to the BCC and face-centered cubic (FCC) phase, as depicted in Figure 2 and Figure 3. The formation of the FCC phase is ascribed to the larger atomic radii of Ti than Cr, Mn and V. As the Ti content increased, Al and Ti with large atomic radii were mainly distributed to the FCC phase, and BCC-2 phase will be gradually to disappear, as presented in Figure 4. The beginning of the FCC phase led to a decrease in the hardness and strength of these MEAs, as presented in Table 1. In Figure 5 and Table 1, the high elastic modulus of Cr (~280 GPa) resulted in high hardness, high compressive yield strength, and poor ductility. Mn addition strongly affected the morphological evolution in the BCC phase. The change of Mn content will affect the morphology of the BCC phase in MEA, resulting in a change in strength, as detailed in Figure 6 and Table 1. Finally, the addition of V increased the fraction of the BCC phase of the MEAs; their compressive yield strength is presented in Figure 7 and Table 1.

**Figure 8:**
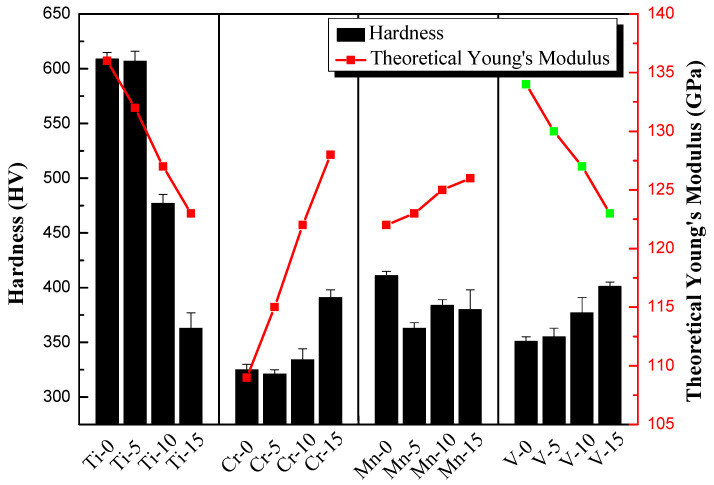
The relationship between hardness and theoretical Young’s modulus of Al_50_–Ti–Cr–Mn–V alloys.

From the previous experimental results, several factors affecting the mechanical properties of the alloy can be observed. In hardness results, the theoretical Young’s modulus has a great influence on the hardness of Al_50_–Ti–Cr–Mn–V alloys. With the addition of high Young’s modulus element like Cr, the theoretical Young’s modulus increases and the hardness of the MEAs will increase. On the other hand, the Ti content increase will effectively reduce the hardness of the alloys, as presented in Figure 8. In tensile testing results, not only theoretical Young’s modulus but also the theoretical atom size difference and the fraction of BCC phase will affect the yielding strength and ductility of the alloys, as presented in Table 2. It can be found that despite the increase of Mn content, there is little effect on the theoretical Young’s modulus and the strength of the alloy still changes significantly. It is believed that the reason for this is related to the variation of the phase morphology. It has been suggested that the theoretical atom size difference of the alloy will led to the variation of the phase morphology. Therefore, the manipulation of the phase morphology can affect the mechanical properties. This phenomenon can also be observed when the increase of Cr content in MEAs. Due to the difference in the atomic radius of MEAs increases obviously with the addition of Cr, the morphology of the phase is refined. Combined with the characteristics of high Young’s modulus, the strength of the MEAs are enhanced significantly. In addition, it can be noticed that the fraction of BCC phase plays an important role in mechanical properties of MEAs. It is well-known that the BCC phase is harder and more brittle than the FCC phase. With the fraction of BCC phase increase, the strength will be enhanced. This phenomenon can also explain why Al_50_(CrMnV)_50_ and Al_50_(CrMnV)_45_Ti_5_ alloys are too brittle to be cast successfully. Despite the theoretical Young’s modulus decrease as the V content increases, the hardness of the MEAs will increase with the V adding due to the fraction of BCC phase increase. Therefore, it is proved that the increase of the ratio of the BCC phase is more dominant than Young’s modulus in improving the mechanical properties of the MEAs.

In summary, High Ti content in Al_50_–Ti–Cr–Mn–V MEAs can lead to a dual phase (BCC + FCC), and the addition of V and Cr can induce high strength. Additionally, the morphology of the BCC phase can be manipulated by adding a moderate Mn ratio. Notably, the effect of the addition of V on the mechanical compressive properties of Al_50_(TiCrMn)_50-x_V_x_ MEAs was significant; the compressive strength of MEAs increased from 1415 MPa to 1833 MPa by adding element V to Al_50_(TiCrMn)_50_ to produce Al_50_(TiCrMn)_45_V_5_. Therefore, fine-tuning V concentrations in Al_50_–Ti–Cr–Mn–V MEAs represents the key method for obtaining favorable mechanical compressive properties. Two approaches for fine-tuning V in MEAs are discussed in detail.

### 3.3. Al_50_(Ti_2_Cr_1_Mn_2_)_50-x_V_x_ Series

**Figure 9:**
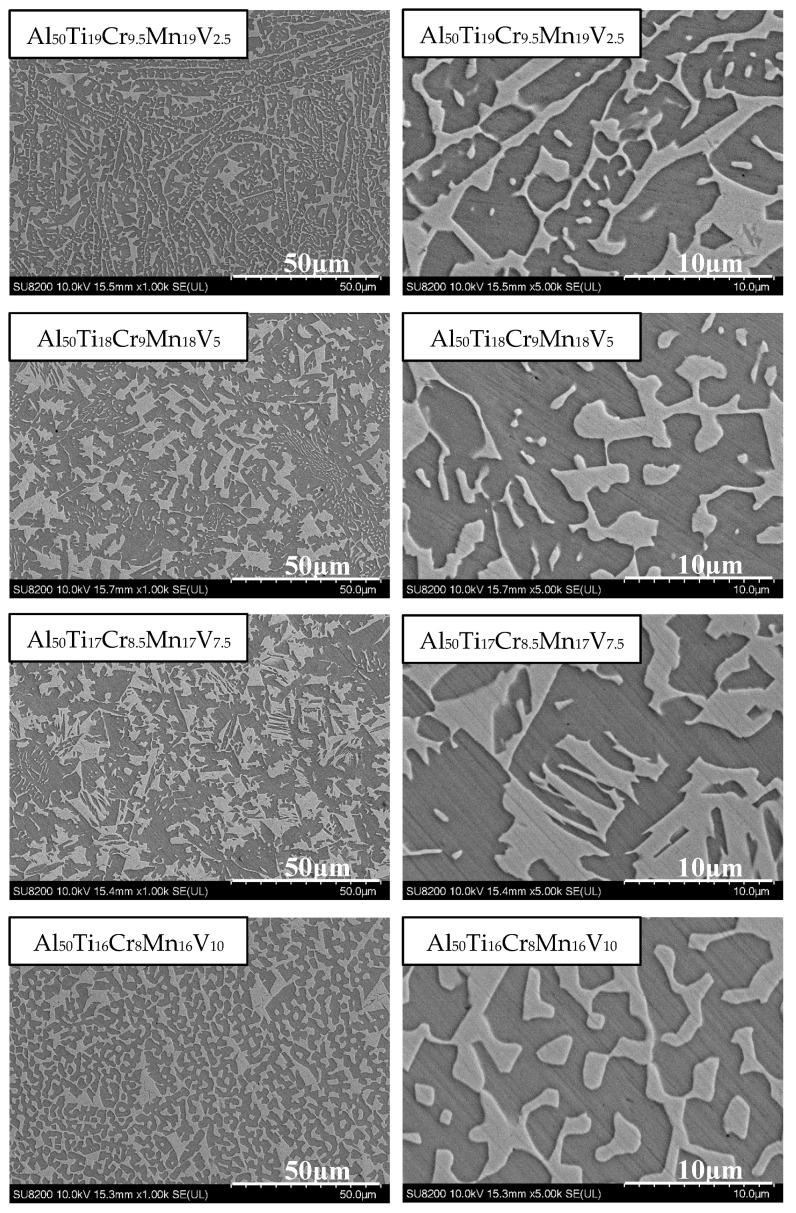
SEM images of the Al_50_(Ti_2_Cr_1_Mn_2_)_50-x_V_x_ (x = 2.5, 5, 7.5, 10) alloys.

Al_50_(Ti_2_Cr_1_Mn_2_)_50-x_V_x_ (x = 2.5, 5, 7.5, 10) MEAs were explored. Since Al_50_Ti_20_Cr_10_Mn_20_ exhibited remarkable mechanical properties (1763 MPa, 32%) in prior studies on Al_50_–Ti–Cr–Mn quaternary MEAs [17], the addition of minor V with a fixing ratio of Ti_2_Cr_1_Mn_2_ was further investigated. The phase morphology analysis and compression results are detailed in Figure 9 and Table 3. The addition of V increased the compressive strength, and the plastic strain decreased. The content of Ti simultaneously decreased. These results are consistent with those of other studies. The addition of Mn did not markedly affect the compressive strength of the alloy. However, Cr was expected to reduce the strength when its content decreased, but the content of Cr was likely too low to exhibit an obvious effect. Al_50_Ti_18_Cr_9_Mn_18_V_5_ presented the most superior compressive mechanical properties in the Al_50_(Ti_2_Cr_1_Mn_2_)_50-x_V_x_ series. The yield strength reached 773 MPa, the maximum compressive strength was 1610 MPa, and the compression ratio was 26%. In comparison with Al_50_(TiCrMn)_45_V_5_, despite the similar compression ratio of Al_50_Ti_19_Cr_9.5_Mn_19_V_2.5_ and Al_50_(TiCrMn)_45_V_5_, the compressive strength was reduced by more than 10%.

### 3.4. Al_50_Ti_20_Cr_10_Mn_20-x_V_x_ Series

To further promote the mechanical properties of the Al_50_–Ti–Cr–Mn–V alloy, an alloy system of Al_50_Ti_20_Cr_10_Mn_20-x_V_x_ (x = 2.5, 5, 7.5, 10) was designed. A high content of Ti improved the toughness, and maintaining a certain content of Cr strengthened the alloy.

**Figure 10:**
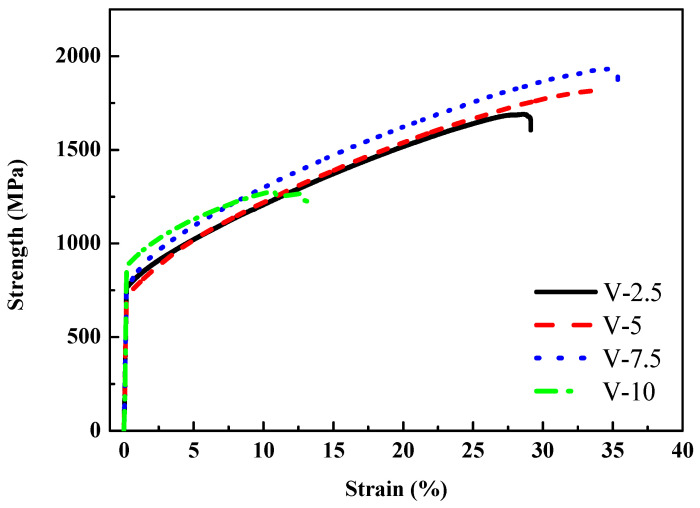
Mechanical compressive stress–strain curves of the Al_50_Ti_20_Cr_10_Mn_20-x_V_x_ (x = 2.5, 5, 7.5, 10) alloys.

In compression testing, compared with Al_50_(Ti_2_Cr_1_Mn_2_)_50-x_V_x_, the compressive strength was enhanced significantly and ductility also improved, as depicted in Figure 10. Al_50_Ti_20_Cr_10_Mn_15_V_5_ exhibited the excellent performance (yield strength was 713 MPa, maximum compressive strength was 1802 MPa, and ductility was 34%). In this case, BCC/FCC ratios in these Al_50_Ti_20_Cr_10_Mn_20-x_V_x_ MEAs are similar. Therefore, significant changes in the morphology of the BCC phases dominate the strength of these investigated MEAs instead of the fraction of BCC phases. Compared with Al_50_(TiCrMn)_45_V_5_ (yield strength was 930 MPa, maximum compressive strength was 1833 MPa, and ductility was 26%) in other studies, and despite the slight reduction in compressive strength, the ductility of the alloy increased significantly and the toughness was enhanced (as presented in Table 3). Although Al_50_Ti_20_Cr_10_Mn_12.5_V_7.5_ possessed higher compressive strength than Al_50_Ti_20_Cr_10_Mn_15_V_5_ (as detailed in Table 3), the deviation in the compressive test results of Al_50_Ti_20_Cr_10_Mn_12.5_V_7.5_ was larger than in those of Al_50_Ti_20_Cr_10_Mn_15_V_5_. Therefore, Al_50_Ti_20_Cr_10_Mn_15_V_5_ is the most promising combination for Al_50_–Ti–Cr–Mn–V MEAs.

**Figure 11:**
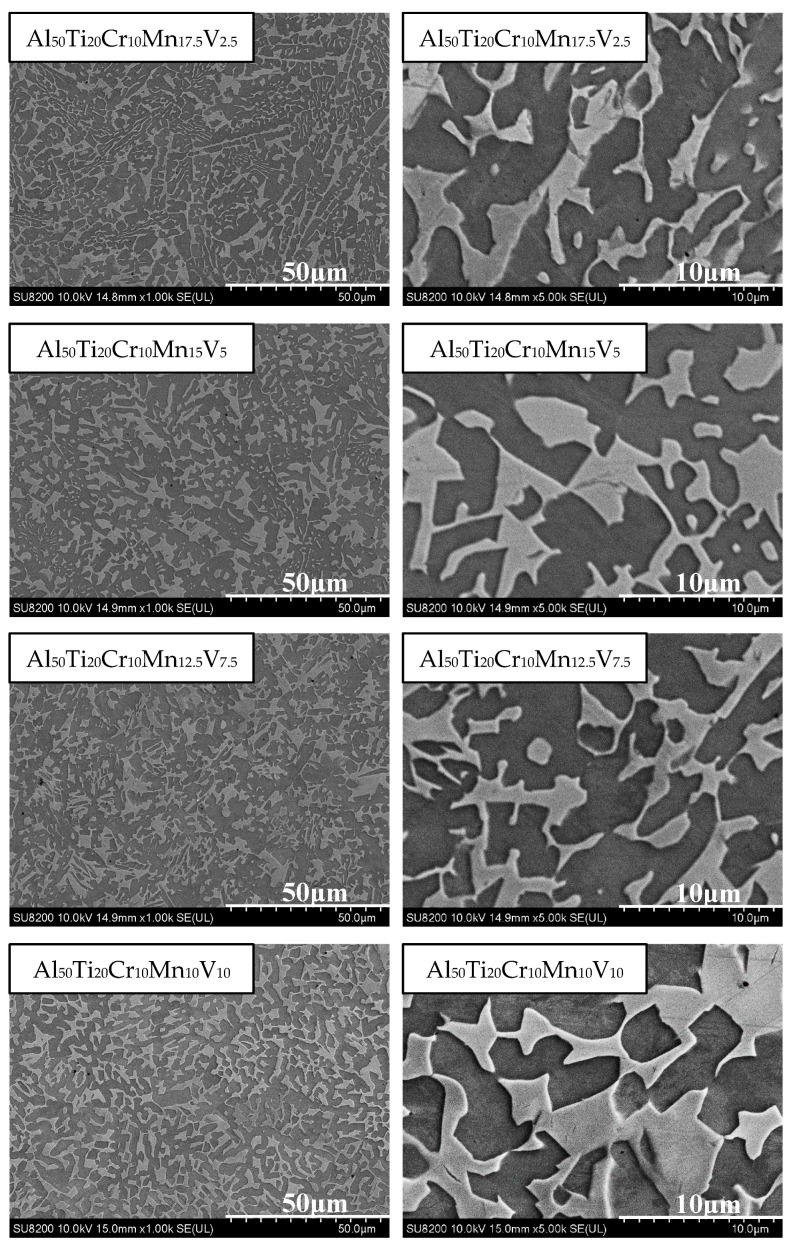
SEM images of the Al_50_Ti_20_Cr_10_Mn_20-x_V_x_ (x = 2.5, 5, 7.5, 10) alloys.

The SEM analysis is illustrated in Figure 11. In Al_50_Ti_20_Cr_10_Mn_17.5_V_2.5_, the morphology presented as a finer dual-phase structure, differing from the morphology of Al_50_Ti_20_Cr_10_Mn_15_V_5_ and Al_50_Ti_20_Cr_10_Mn_12.5_V_7.5_, which presented in the shape of a bamboo leaf. When the V content was increased to 15%, the phase became thicker and the morphology of Al_50_Ti_20_Cr_10_Mn_10_V_10_ changed into an island shape.

### 3.5. Annealing Treatment on the Al_50_Ti_20_Cr_10_Mn_15_V_5_ Series

As mentioned above, the mechanical properties of the MEAs are linked to the morphology of the dual phases. To improve the mechanical properties through manipulation of the morphology of the BCC and FCC phases, annealing treatments were conducted on the promising Al_50_Ti_20_Cr_10_Mn_15_V_5_ MEA. Annealing treatment was performed at 1000 °C for varying lengths of time, followed by air-cooling or oil-quenching processes in a high vacuum atmosphere.

**Figure 12:**
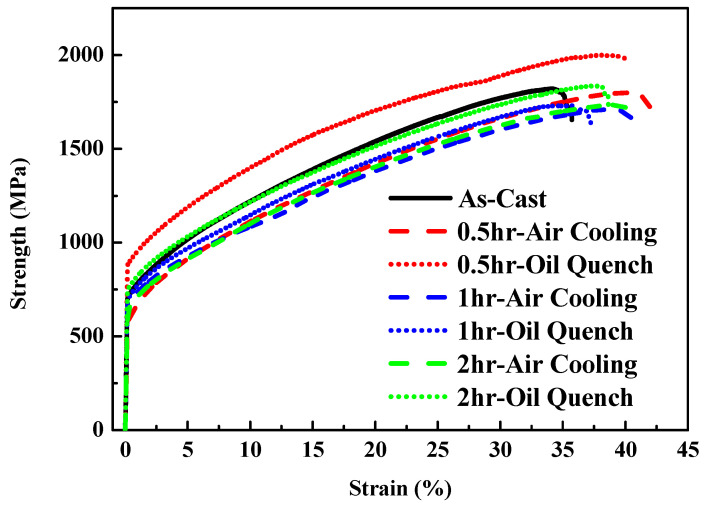
Mechanical compressive stress–strain curves of the Al_50_Ti_20_Cr_10_Mn_15_V_5_ MEAs annealed at 1000 °C for 0.5, 1, and 2 h, followed by air-cooling and oil-quenching.

**Figure 13:**
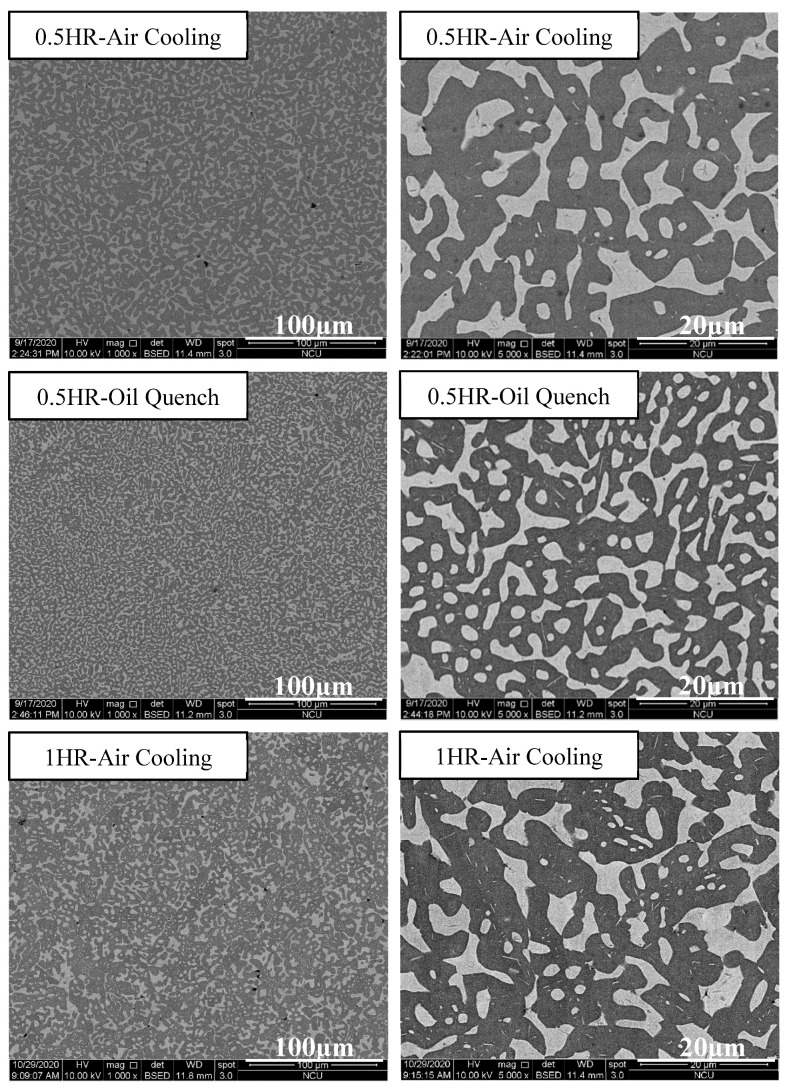

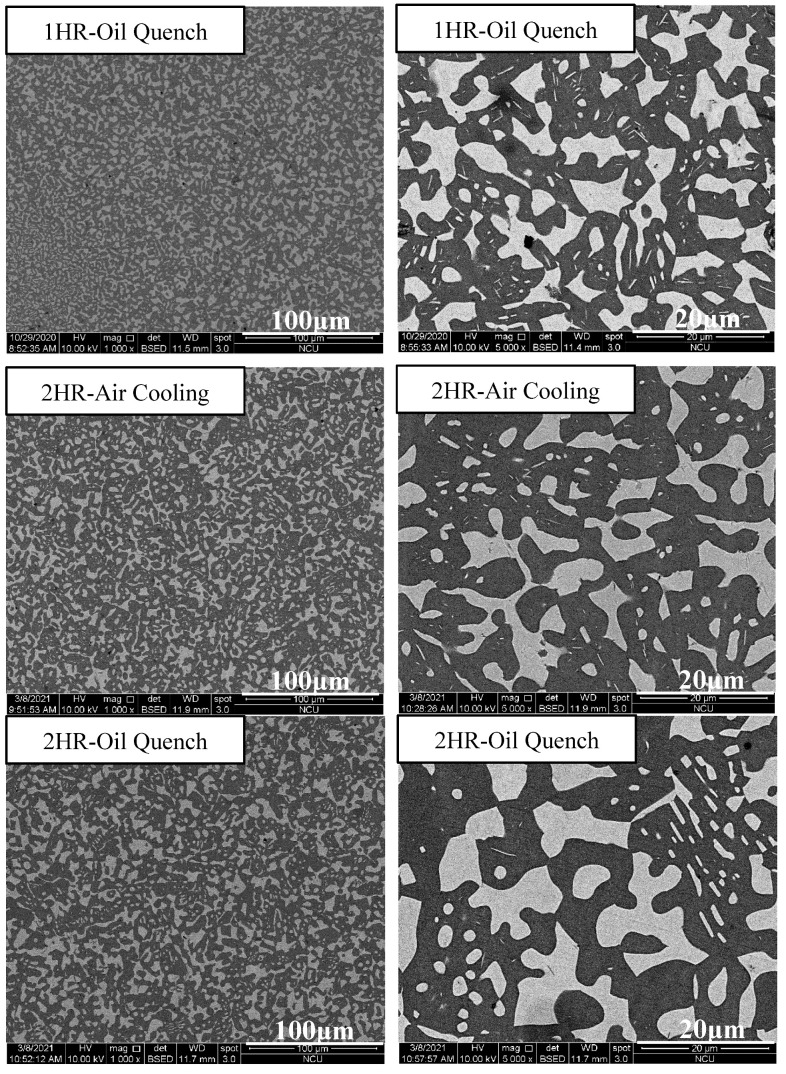
SEM images of the Al_50_Ti_20_Cr_10_Mn_15_V_5_ MEAs annealed at 1000 °C for 0.5, 1, and 2 h, followed by air-cooling and oil-quenching.

Figure 12 depicts the strength–strain curves of Al_50_Ti_20_Cr_10_Mn_15_V_5_ annealed at 1000 °C for 0.5, 1, and 2 h, followed by air-cooling and oil-quenching. Through the annealing treatment, the plastic strain of Al_50_Ti_20_Cr_10_Mn_15_V_5_ increased slightly. Notably, the annealed and oil-quenched Al_50_Ti_20_Cr_10_Mn_15_V_5_ exhibited extraordinary compressive strength and ductility (1966 MPa and 40%). The compressive test results and phase fraction are summarized in Table 4. The oil-quenching process significantly improved the compressive strength of the MEAs through fraction evolution and the morphology of the BCC phase, as presented in Figure 13. The oil-quenching process induced higher strength in the MEAs compared with the air-cooling process. It supposed that the increase of BCC fraction which was attributed to rapid cooling rates led to increase in strength. However, with increased annealing time, the phase morphology become more rounded and the strength decreased slightly. For improving the mechanical properties through manipulation of phase morphology, the quenching approach was more effective than annealing time in this study.

Compared with the papers published in recent years on the study of MEAs, several of them have conducted in-depth discussions on their density and mechanical properties [10,18,19,20]. Among them, the casting Al_70_Cu_5_Mg_5_Si_10_Zn_5_X_5_ systems MEAs studied by Jon et al. [18] were the most comparable to this study due to the main element of Al. Despite the fact that the density of Al_70_Cr_5_Cu_5_Mg_5_Si_10_Zn_5_ is lower due to the relatively high proportion of light elements, the high Al content and the formation of intermetallic compounds make the performace of strength and ductility of Al_70_Cr_5_Cu_5_Mg_5_Si_10_Zn_5_ much worse than Al_50_Ti_20_Cr_10_Mn_15_V_5_. In particular, the specific compressive strength of Al_50_Ti_20_Cr_10_Mn_15_V_5_ was attended to 452 MPa·g/cm^3^, which is more than twice that of Al_70_Cr_5_Cu_5_Mg_5_Si_10_Zn_5_ with 199 MPa·g/cm^3^. On the other hand, the Al_15_(CuFeMn)_85_ MEAs proposed by Jongun et al. also represents a very comparable study [20]. Through the heat treatment, the strength and ductility of Al_15_(CuFeMn)_85_ presented exceptional performance. However, due to the large proportion of heavy elements in MEAs, the density of the material had been greatly increased, making the specific strength of the alloy only 143.87, which was less than one-third of Al_50_Ti_20_Cr_10_Mn_15_V_5_. In addition, Bingbing et al. proposed the MEAs about the addition of Zn and Mg in 5083 aluminum alloy which considered the strengthening mechanisms of solid solution strengthening and grain boundary strengthening. Moreover, the yield strength of the MEAs was increased from 86 MPa to 423 MPa while maintaining a certain ductility, which means that the strength of the MEAs was effectively improved through the strengthening mechanism. This characteristic will be used to explore and improve the mechanical properties of subsequent MEAs.

## 4. Conclusions

A novel dual-phase lightweight nonequiatiomic Al_50_–Ti–Cr–Mn–V quinary MEA system was developed. Based on the Al_50_–Ti–Cr–Mn–V quinary alloy, the ratio of each element was adjusted and the influence of each element was analyzed. According to the experiment results, conclusions about the microstructure evolution and mechanical properties of the alloy system can be summarized as follows:

1. By maintaining a 50% Al atomic ratio, all designed alloys could achieve the low density (<5 g/cm^3^) target.
2. Most of the MEAs exhibited a dual-phase (FCC + BCC) structure. Cr, Mn, and V with their small atomic radii were mainly distributed in the BCC phase, and Al and Ti with their large atomic radii were mainly distributed in the FCC phase. However, if the Ti element concentration was not sufficient in the alloy, the structure converted to dual BCC phases.
3. Increasing the Ti content softened the MEAs. Different Mn concentrations affected the shape of the BCC phase, resulting in changes to the mechanical properties. An increase in Cr and V contents significantly increased the hardness and strength of the alloy.
4. The fraction and morphology of the BCC phase played a key role in the resultant mechanical properties. By increasing the fraction of the BCC phase, the strength of the MEAs could be enhanced. In addition, the MEAs with a round-shaped BCC phase possessed higher ductility than those with a sharp-edged phase.
5. Concerning the Al_50_–Ti–Cr–Mn–V MEA systems, the Al_50_Ti_20_Cr_10_Mn_15_V_5_ MEA exhibited the best mechanical properties after annealing (at 1000 °C for 0.5 h) and oil-quenching, with an 802 MPa yield strength, 1966 MPa compressive strength, and 40% plastic strain. The specific strength-to-density ratio could reach 452 MPa·g/cm^3^.

## Figures and Tables

**Table 1:** Mechanical compressive properties of the Al_50_–Ti–Cr–Mn–V alloys.

Constituent	Hardness (HV)	Yield Strength (MPa)	Ultimate Strength (MPa)	Ductility (%)
Al_50_(CrMnV)_50_	609 ± 6	Cannot be produced		
Al_50_(CrMnV)_45_Ti_5_	607 ± 9			
Al_50_(CrMnV)_40_Ti_10_	477 ± 8	1164 ± 58	1182 ± 40	1 ± 1
Al_50_(CrMnV)_35_Ti_15_	363 ± 14	870 ± 95	1171 ± 388	11 ± 10
Al_50_(TiMnV)_50_	335 ± 5	756 ± 30	1349 ± 131	18 ± 3
Al_50_(TiMnV)_45_Cr_5_	321 ± 4	851 ± 18	1293 ± 113	17 ± 5
Al_50_(TiMnV)_40_Cr_10_	334 ± 10	977 ± 50	1457 ± 110	16 ± 3
Al_50_(TiMnV)_35_Cr_15_	391 ± 7	989 ± 123	1042 ± 109	1 ± 1
Al_50_(TiCrV)_50_	411 ± 4	1258 ± 148	1752 ± 118	18 ± 2
Al_50_(TiCrV)_45_Mn_5_	363 ± 5	1004 ± 15	1430 ± 144	14 ± 3
Al_50_(TiCrV)_40_Mn_10_	384 ± 5	861 ± 63	958 ± 136	5 ± 4
Al_50_(TiCrV)_35_Mn_15_	380 ± 18	1005 ± 27	1294 ± 88	9 ± 1
Al_50_(TiCrMn)_50_	351 ± 4	737 ± 45	1415 ± 42	17 ± 1
Al_50_(TiCrMn)_45_V_5_	355 ± 8	930 ± 22	1833 ± 165	26 ± 3
Al_50_(TiCrMn)_40_V_10_	377 ± 14	948 ± 27	1424 ± 198	15 ± 8
Al_50_(TiCrMn)_35_V_15_	401 ± 4	905 ± 33	964 ± 26	3 ± 1

**Table 2:** Theoretical Young’s modulus, atom size difference and the phase fraction of the Al_50_–Ti–Cr–Mn–V alloys.

Constituent	Young’s Modulus (GPa)	Atom Size Difference (pm)	FCC/BCC-1/BCC-2
Al_50_(CrMnV)_50_	136	5.12	0/28/72
Al_50_(CrMnV)_45_Ti_5_	132	5.14	0/49/51
Al_50_(CrMnV)_40_Ti_10_	127	5.09	41/25/34
Al_50_(CrMnV)_35_Ti_15_	123	4.98	66/34/0
Al_50_(TiMnV)_50_	109	3.72	61/39/0
Al_50_(TiMnV)_45_Cr_5_	115	4.33	57/43/0
Al_50_(TiMnV)_40_Cr_10_	122	4.82	68/32/0
Al_50_(TiMnV)_35_Cr_15_	128	5.24	68/32/0
Al_50_(TiCrV)_50_	122	5.57	57/43/0
Al_50_(TiCrV)_45_Mn_5_	123	5.36	60/40/0
Al_50_(TiCrV)_40_Mn_10_	125	5.15	53/47/0
Al_50_(TiCrV)_35_Mn_15_	126	4.93	59/41/0
Al_50_(TiCrMn)_50_	134	5.24	71/29/0
Al_50_(TiCrMn)_45_V_5_	130	5.16	61/39/0
Al_50_(TiCrMn)_40_V_10_	127	5.08	53/47/0
Al_50_(TiCrMn)_35_V_15_	123	5.00	57/43/0

**Table 3:** Mechanical compressive properties of the Al_50_(Ti_2_Cr_1_Mn_2_)_50-x_V_x_ (x = 2.5, 5, 7.5, 10) and Al_50_Ti_20_Cr_10_Mn_20-x_V_x_ (x = 2.5, 5, 7.5, 10) alloys.

Constituent	FCC/BCC	Yield Strength (MPa)	Ultimate Strength (MPa)	Ductility (%)
Al_50_Ti_19_Cr_9.5_Mn_19_V_2.5_	69/31	727 ± 23	1484 ± 140	25 ± 5
Al_50_Ti_18_Cr_9_Mn_18_V_5_	68/32	773 ± 32	1610 ± 45	26 ± 1
Al_50_Ti_17_Cr_8.5_Mn_17_V_7.5_	65/35	791 ± 40	1291 ± 346	20 ± 7
Al_50_Ti_16_Cr_8_Mn_16_V_10_	62/38	865 ± 83	1473 ± 59	20 ± 1
Al_50_Ti_20_Cr_10_Mn_17.5_V_2.5_	71/28	743 ± 17	1576 ± 77	27 ± 2
Al_50_Ti_20_Cr_10_Mn_15_V_5_	69/31	713 ± 9	1802 ± 22	34 ± 1
Al_50_Ti_20_Cr_10_Mn_12.5_V_7.5_	67/33	763 ± 6	1882 ± 35	33 ± 3
Al_50_Ti_20_Cr_10_Mn_10_V_10_	66/34	876 ± 33	1266 ± 6	14 ± 4

**Table 4:** Mechanical compressive properties of the Al_50_Ti_20_Cr_10_Mn_15_V_5_ MEAs annealed at 1000 °C for 0.5, 1, and 2 h, followed by air-cooling and oil-quenching.

Constituent	FCC/BCC	Yield Strength (MPa)	Ultimate Strength (MPa)	Ductility (%)
0.5 h-air cooling	72/28	659 ± 83	1779 ± 13	34 ± 5
0.5 h-oil quenching	68/32	802 ± 47	1966 ± 27	40 ± 1
1 h-air cooling	71/29	659 ± 13	1696 ± 17	38 ± 2
1 h-oil quenching	69/31	714 ± 14	1720 ± 12	36 ± 2
2 h-air cooling	71/29	671 ± 31	1675 ± 57	35 ± 5
2 h-oil quenching	67/33	744 ± 18	1724 ± 79	33 ± 4

## Data Availability

The data presented in this study are available on request from the corresponding author.
